# The impact of stigmatization of psoriasis, atopic dermatitis and mastocytosis in different areas of life—A qualitative interview study

**DOI:** 10.1002/ski2.62

**Published:** 2021-07-29

**Authors:** P. Heim‐Ohmayer, A. Freiberger, M. Gedik, J. Beckmann, S. Ziehfreund, A. Zink, W. Hähl, M. C. Schielein

**Affiliations:** ^1^ Department of Sport and Health Sciences Technical University of Munich Munich Germany; ^2^ School of Human Movement and Nutrition Sciences The University of Queensland Brisbane Queensland Australia; ^3^ Department of Dermatology and Allergy School of Medicine Technical University of Munich Munich Germany; ^4^ Unit of Dermatology and Venereology Department of Medicine Karolinska University Hospital Karolinska Institutet Solna Sweden

## Abstract

**Background:**

Stereotypes and false assumptions about chronic and visible skin diseases can determine the behaviour towards affected individuals and result in stigmatization or discrimination.

**Objective:**

The aim of this study was to analyze the perceived disease‐related stigmatization of individuals with psoriasis, atopic dermatitis (AD) or mastocytosis. The study also aims to broaden people‐centred knowledge of the effects of stigmatization in different areas of life, namely in everyday life, at work, in sports and in relationships.

**Methods:**

Qualitative in‐depth semi‐structured interviews were conducted among individuals with either psoriasis, AD or mastocytosis. Participants were recruited via self‐help networks and were asked to express their experience of stigmatization in different areas of life. All interviews were audio recorded, transcribed verbatim and evaluated based on Mayring's content analysis.

**Results:**

In total, 24 individuals aged 19–79 years and living in Germany were included in the study—eight for each disease. Stigmatization was experienced in all three diseases in all mentioned areas of life as well as in interaction with medical professionals. Self‐exclusion, negative self‐perception and negative behaviour of others were the most frequent experiences with stigmatization.

**Conclusion:**

Stigmatization, both internal and external, is an important factor contributing to the mental burden of people with chronic skin diseases. More research is needed to gain deeper insight into stigmatization and its psychological burden in various contexts to enhance people‐centred care in chronic skin diseases.

## Graphical abstract

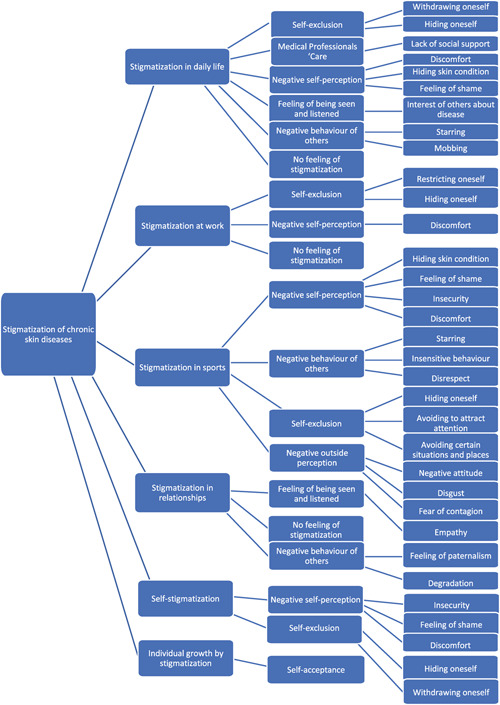

1

**What's already known about this topic?**

- Individuals with chronic skin diseases show impairments in quality of life, including mental health and emotional consequences
- One possible reason for mental impairment is stigmatization

**What does this study add?**

- This study examines stigmatization of individuals suffering from psoriasis, atopic dermatitis or mastocytosis
- Stigmatization was stratified for different areas of life, namely in everyday life, at work, in sports and in relationships

**What are the clinical implications of this work?**

- More research is needed to better understand affected individuals and to develop questionnaires for the quantification of stigmatization of chronic skin diseases
- Next steps should also include the development of suitable help programs for affected individuals and their environment to enhance people‐centred care in chronic skin diseases

## 2 INTRODUCTION

Skin diseases are common and show an overall point prevalence between 1.4% and 64.5% across Europe.^1^, ^2^ Severity, duration and localization vary widely.^3^, ^4^, ^5^ Individuals with chronic and inflammatory skin diseases show impairments in quality of life (QoL), including mental health and emotional consequences.^6^, ^7^ Accordingly, these individuals often suffer from depression and anxiety.^8^ One potential reason for adverse mental health outcomes and an increased risk for mental disorders is stigmatization.^9^, ^10^, ^11^, ^12^ Due to limited societal acceptance, affected individuals often seclude themselves from everyday life and therefore may be more susceptible to mental comorbidities.^12^ While a wide range of research on mental comorbidities of chronic and often visible skin diseases exists,^13^, ^14^, ^15^ the current research on stigmatization is limited. A recent review therefore suggested considering a variety of visible skin diseases as the particular dimensions of stigmatization and its impact may vary throughout different diseases.^16^

The high mental burden caused by chronic skin diseases prompted the World Health Organization (WHO) to demand a transition to people‐centred care in its Global Report on Psoriasis. The people‐centred care approach requires health care systems to consider the full spectrum of an individual's needs, including not only issues related to diseases but also other concerns related to health and well‐being.^17^ For the investigation of stigmatization, ‘one fits all’ approaches are often applied,^18^ meaning that analyses of stigmatization rely on questionnaires that can be applied to ‘all’ types of diseases without precise differentiation. However, as skin diseases are highly diverse, this ‘one fits all’ approach is not suitable to analyze stigmatization of chronic skin diseases in order to tailor health services to individual needs.^19^

Psoriasis, atopic dermatitis (AD) and cutaneous mastocytosis (CM) are prime examples of visible chronic skin disease. The prevalence for psoriasis and AD in adults in Europe is high (2%–2.5% and 4.4%, respectively),^3^, ^20^ while mastocytosis is less prevalent and most likely underdiagnosed.^21^, ^22^ Moreover, these diseases are characterized by different levels of evidence and by variations in their manifestation.^3^, ^20^, ^21^ Psoriasis is characterized by affliction of the skin, nails or joints, and the main symptoms include redness, plaques and scaling of the skin, which are preferentially located on the elbows, knees, sacrum, coccyx, navel and scalp.^3^ AD is a multifaceted, chronic relapsing inflammatory skin disease with exacerbations and remissions of eczematous skin that is characterized by inflammation, pruritus, excoriations and dryness.^23^ The clinical features of mastocytosis include flushing, pruritus, abdominal pain, diarrhoea, hypotension, syncope and musculoskeletal pain.^24^ It can be classified as cutaneous (affecting only the skin) or systemic (affecting one or more internal organs) mastocytosis.^25^

These three skin diseases are well suited for a thorough investigation of the experienced stigmatization of persons with chronic skin diseases beyond that of previous studies, which focused predominantly on stigmatization of singular skin diseases only. Therefore, the aim of this study was to gain an insight in the differential, perceived disease‐related stigmatization among individuals with psoriasis, AD or mastocytosis and expand knowledge about its effects and impact on different areas of life, namely in everyday life, at work, in sports and in relationships.

## 3 METHODS

### 3.1 Data collection

A qualitative study using in‐depth semi‐structured interviews was conducted among individuals with psoriasis, AD or mastocytosis. The ‘Consolidated criteria for reporting qualitative research (COREQ)’‐ checklist was applied to develop the study and draft the manuscript.^26^ Interviews ranged between 30 and 40 min in length. Each patient group, psoriasis, AD or mastocytosis, was interviewed by one of three interviewers (each female with a B.Sc. in Health Sciences) between November and December 2020 in Germany. Individuals were aware of the research goals and researcher's characteristics (name and interest in the research topic). Interviewers were not familiar with the participants prior to study initiation. A previously generated topic guide including open‐ended questions was used. The topic guide was pre‐tested for each patient group to check for comprehension and the timeframe. No adjustments had to be made, and thus the interviews were included in the analysis. The interview guide included warm‐up questions aiming to establish a good rapport with the participants as well as a short inquiry if there were still remaining questions concerning the procedure of the study. The next part comprised several main open‐ended questions that helped define the areas to be explored. Probing and detail questions were also used to elicit more detailed responses to key questions such as ‘Can you tell me more about this situation?’ For the topic guide, see Table 1.

**TABLE 1:** Topic, main questions and detail questions

Topic	Main questions	Detail questions
Patient journey^a^	‘How was your patient journey?’	‘When did you get the diagnosis and from whom?’
	‘Which experiences have you had?’	‘Can you tell me more about your journey?’
Disease description	‘Can you describe your disease and its clinical picture?’	‘Are there factors that trigger an exacerbation of the disease?’
		‘Can you tell me more about your symptoms?’
Stigmatization	‘What experiences have you had with stigmatization in general?’	‘How did you feel in situations where you were stigmatized?’
	‘What experiences with stigmatization have you had in situations of everyday life—especially at work, in sport and in relationships?’	‘Can you tell me more about these situations?’

^a^ Was conducted but not evaluated in this paper.

The topic guide was developed in cooperation with dermatologists and scientific assistants based on relevant literature^27^ using the Helfferich principle.^28^ Field notes were taken following each interview. Due to the COVID‐19 pandemic, all interviews were conducted online, audio‐recorded and transcribed verbatim. Both interviewers and participants were at home without any disturbances. No interview had to be repeated. Data collection proceeded until data saturation was achieved.

### 3.2 Participant recruitment

For the recruitment of participants, national patient networks and online self‐help groups (Psoriasis‐Netz, Deutscher Neurodermitisbund e.V., Deutscher Allergie‐ und Asthma‐Bund e.V., Mastozytose Selbsthilfe Netzwerk e.V., Mastozytose e.V.) were contacted. Participants were informed about the study via e‐mail and posts on different social media platforms and websites of the patient networks and groups. A consecutive sampling method was used. Each participant who contacted the research team and met the inclusion criteria was involved. Study information and contact details of the study team were distributed. Participants had to be 18 years or older, German speaking, and have either psoriasis, AD or mastocytosis as diagnosed by a dermatologist. Individuals with any combination of the three investigated diseases were excluded due to possible confounding of the data. Written informed consent was obtained from all participants prior to each interview. The study was conducted in accordance with the declaration of Helsinki and was reviewed and approved by the Ethics Committee at the Technical University Munich (reference 534/20 S).

### 3.3 Data analysis

Data analysis was performed using the qualitative content analysis by Mayring.^29^ Data saturation was reached after eight interviews for each disease, as no new information was given concerning the experienced stigmatization in the different areas of life. An interpretative phenomenological analysis was used due to its combination of psychological, interpretative and idiographic components.^30^ Categories were generated using an inductive‐deductive approach; category development was guided by a systematic reduction process during data analysis. The interviews were evaluated by the respective interviewer. After re‐reading the interview transcripts, text sequences were placed in suitable categories, and thereby codes were generated.^29^ Uncertainties concerning the coding scheme were discussed by the researchers and homogeneously adjusted.

## 4 RESULTS

This study comprised semi‐structured interviews of 24 individuals suffering from psoriasis (*n* = 8), AD (*n* = 8), or mastocytosis (*n* = 8). The majority of the sample was female (*n* = 16, 66.7%), and participants were aged between 19 and 79 years. The mean age was 44.4 ± 16.0 years. For detailed sample characteristics, see Tables 2 and 3.

**TABLE 2:** Distribution of gender and age in the three diseases

Disease		Gender (*n*)	Age range [years]	Mean age [years]	SD age [years]
Psoriasis	Female	3	40–79	53.4	14.2
	Male	5			
AD	Female	5	19–60	39.8	17.4
	Male	3			
Mastocytosis	Female	8	22–61	40.1	14.1
	Male	0			

Abbreviations: AD, atopic dermatitis; SD, standard deviation.

**TABLE 3:** Affected areas and age of onset of the participants

Participant	Sex	Age	Disease	Particularly affected areas^a^	Onset of first symptoms^b^
1	M	45	PSO with Arthritis	Elbow, joints	Adulthood (∼30 years)
2	F	64	PSO	N/A	Childhood (3 years)
3	F	40	PSO with Arthritis	Scalp, toenails, elbows, belly, belly button, coccyx, upper back, knees, shins, thighs, palms, soles, partly genital area	Childhood (9 years)
4	M	40	PSO	N/A	Adulthood (∼20 years)
5	M	41	PSO	Face, elbow, shins, knees	Adolescence (∼17 years)
6	M	60	PSO with Arthritis	Joints of knees and toes, shoulders, and fingers	Adulthood (∼34 years)
7	F	58	PSO	Elbow, knee, legs, arms	Adolescence (17 years)
8	M	79	PSO	Lower legs, knee, sometimes behind the ear	Adulthood (∼65 years)
9	M	60	AD	Face, crook of the arms, legs	Within first 6 months
10	F	21	AD	N/A	Within first 6 months
11	F	30	AD	N/A	Within first 6 months
12	F	54	AD	N/A	Within first 6 months
13	F	50	AD	N/A	Within first 6 months
14	F	26	AD with Asthma	Face, crook of the arm, back of the knees	Infant
15	M	58	AD with Asthma	N/A	Infant
16	M	19	AD	Hands	Early childhood
17	F	44	CM (urticaria pigmentosa)	Buttocks, upper arm	Adulthood (∼30 years)
18	F	61	CM	Thighs, belly, back	Adolescence (∼15 years)
19	F	43	CM	Whole body	Adulthood (Mid‐twenties)
20	F	36	CM	N/A	Adulthood (Mid‐twenties)
21	F	58	CM and SM	Legs, belly, arms, back (around 35% of the skin is covered)	Adulthood (∼46 years)
22	F	27	CM	Face, back of the feet, few on belly	Adulthood (Mid‐twenties)
23	F	30	CM	Thighs, torso, back	Adulthood (Mid‐twenties)
24	F	22	CM (urticaria pigmentosa)	N/A	Infant

*Note:* Information was extracted from interviews. No assurance of completeness.

Abbreviations: AD, atopic dermatitis; CM, cutaneous mastocytosis; PSO, psoriasis; SM, systemic mastocytosis.

^a^ Variable due to qualitative interviews.

^b^ Approximal age ranges.

**FIGURE 1:**
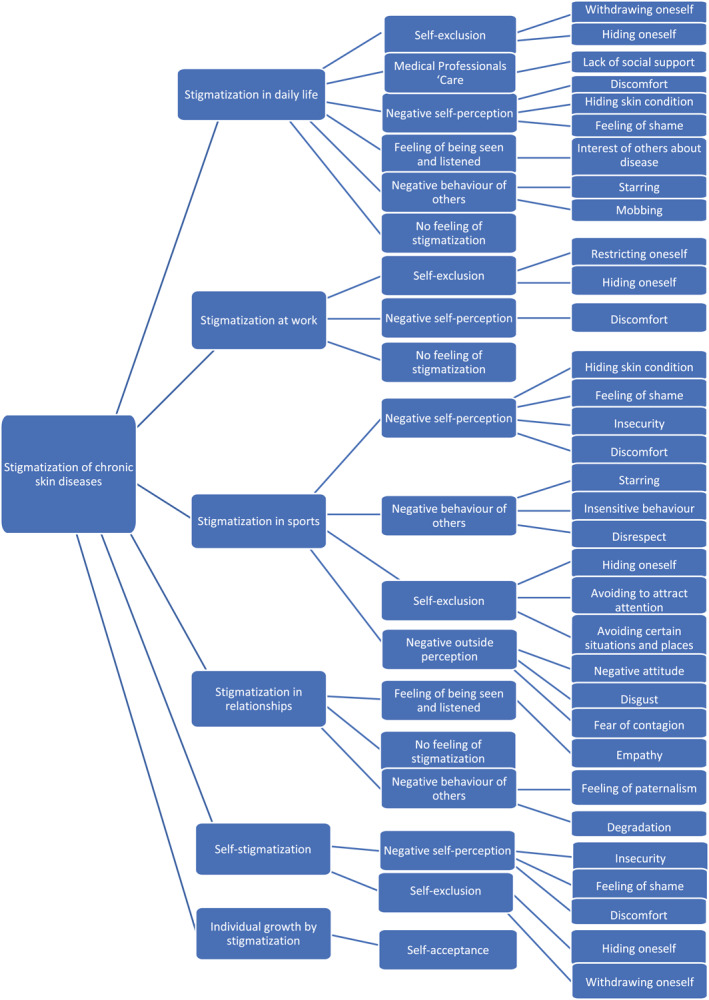
Tree of categories, sub‐categories and codes

Since the focus of this qualitative study is stigmatization in different areas of life, four main categories were initially established: stigmatization in daily life, at work, in sports and in relationships. During data analysis, two additional main categories were added, which were also mentioned by participants: the occurrence of self‐stigmatization and individual growth by stigmatization. More than 40 codes were extracted from the interviews in data analysis. During the process, these codes were summarized into eight subcategories (Figure 1): self‐exclusion, negative self‐perception, self‐acceptance, negative outside perception, feeling of being seen and listened, negative behaviour of others, lack of social support, and no feeling of stigmatization. All generated subcategories were examined in each main category.

### 4.1 Stigmatization in everyday life

Several participants reported negative self‐perception in everyday life. Frequently mentioned reasons were discomfort, hiding skin irritation and feelings of shame. A participant said: ‘[…] I really feel uncomfortable. So, when I put on a dress in summer or something, I always make sure that it covers my thighs’ (CM, female (f), 27). Additionally, several participants, but none with mastocytosis, expressed experiencing self‐exclusion, mostly by withdrawing or hiding oneself. One participant said: ‘And that's why I kind of stayed away from those things. So, I took myself out of the game a little bit’ (AD, f, 30).

Many participants for all diseases experienced negative behaviour from others in their past. This was mostly by staring but also through more extreme forms including bullying: ‘She [the acquaintance's daughter] bullied me all the time. That's when I really picked my skin off. It was really bad. I remember, I was standing in bed and I pulled my skin off. […] ‘What do you look like? Yuck.’ Totally awful. At school, even in sports, that still played a role’ (AD, male (m), 58).

However, some participants stated that they did not feel stigmatization when looked at: ‘No, no, I don't have any [feelings of stigmatization], even in everyday life or something, that people look at me funny or […] that they then find me weird […] no, not at all’ (CM, f, 43).

Participants often felt isolation, lack of interest and misunderstanding from medical professionals regarding the disease. Statements like ‘The doctors dismiss you […] I sometimes felt like a hypochondriac’ (CM, f, 27) and ‘There have been doctors who would have preferred to have touched you with a pair of pliers and who have really made their disgust known’ (psoriasis, f, 40) underline the constant presence of these concerns.

### 4.2 Stigmatization at work

Several participants seemed to isolate themselves at work, mostly by restricting and hiding themselves: ‘Yes, there were some things I could never take part in or which I could blow off right from the start, even later on the job. […] It was not possible with that skin condition. And I also didn't want to present myself there’ (AD, m, 60).

Additionally, some participants seemed to suffer from negative self‐perception at work. Discomfort was one of the main reasons stated by several participants. ‘In the office where I was trained, at the computer there was a black mouse. […] When I touched the mouse and the lady who was sitting next to me, I think she asked herself: What is this? Because the mouse was full of skin’ (AD, f, 50).

Regarding work environments, some participants mentioned not feeling stigmatized. This is supported by statements like ‘Well, […] it is very rare that someone has really noticed it or has addressed it. So, I couldn't say anything specific right now’ (psoriasis, m, 40).

### 4.3 Stigmatization in sports

A number of participants reported a negative perception of themselves due to their skin disease. Some of them tried to hide their skin conditions whenever possible during any kind of physical activity. One participant said: ‘When I go swimming, […] I try to wear not a bikini but a swimsuit. Try to hide my legs a little bit. Yes, it feels unpleasant if other people look at me’ (CM, f, 36). Other participants said they had feelings of shame, insecurity, or discomfort during sports. For some, these feelings were so strong that they quit sports: ‘So, I don't like sports at all. I enjoyed dancing and I would have loved to do classical ballet. But of course, I could not do this […] You cannot run around there with psoriasis. So, this had died for me’ (psoriasis f, 64). Stigmatization can lead to a process of self‐exclusion, which results in avoidance of certain situations such as going to public swimming pools or saunas: ‘It is easy: public swimming pools are not for me. I gave this up really quickly’ (psoriasis, f, 58).

Many participants had negative experiences regarding the behaviour of people around them. This behaviour included staring, insensitive behaviour regarding the skin disease, and in most of the cases disrespect. One participant spoke of her tango dance session: ‘[…] After this one dance session, I had redness and swelling on my cheek and then there are people who laugh about it. And I have to say, I find that hurtful’ (AD, f, 54).

Some participants reported that they experienced negative perception of their disease by others. This was expressed by other people showing negative attitudes towards the skin disease. In extreme cases, people showed disgust or fear of contagion. For example, one person talked about a situation in the gym: ‘I was asked if I had a contagious disease […] I was asked if I was contagious and I even had to show a doctor's certificate that shows that I don't have any contagious disease’ (psoriasis, m, 60).

### 4.4 Stigmatization in relationships

Many participants had positive experiences with their partner's reaction and handling of their disease. They expressed a feeling of being seen and listened to: ‘It is really easy in my relationship. We can talk about it and that is what makes it easy. And yeah, one can handle it really well then’ (AD, f, 21).

Other participants also felt socially supported by their partners: ‘No, no. He knows me. And he loves me with my spots, and he loved me before I had them’ (CM, f, 58). Another positive aspect is that several participants stated that they never had any experiences of stigmatization with partners or potential partners. ‘I've never received any negative feedback, rather only positive feedback […] I've never had any negative experience in my relationship’, said one participant (AD, f, 26).

In contrast, some participants did experience negative reactions from their significant others. These experiences can be divided into feelings of paternalism and degradation. While feelings of paternalism most likely occurred without malicious intentions, degradation was seemingly done purposefully. ‘[…] And then he, my partner, said to me: take a look at yourself. No one wants to touch you because of it’ (psoriasis, f, 40).

### 4.5 Self‐stigmatization

Many participants indicated negative self‐perception. This was mainly expressed through insecurities regarding their own appearance, feelings of shame, attempts at hiding the affected skin, and discomfort. Some participants labelled their disease as not aesthetic: ‘Somehow [pause] I don't think I'm beautiful’ (CM, f, 43). Self‐exclusion was also often reported. Affected participants indicated that they hid themselves to avoid uncomfortable situations. Furthermore, others stated that they even actively withdrew themselves from their environment: ‘You cannot see yourself in the mirror or you want to hide yourself’ (psoriasis, m, 45).

### 4.6 Individual growth by stigmatization

Some participants showed a positive internal change triggered by experienced stigmatization during their lifetime: several participants reported self‐acceptance—mostly expressed through acceptance of the skin condition. Some participants even stated that they perceived with time an increasing internal acceptance of their skin condition: ‘[…] it's just on your body and then you accept it. So, like a scar, like a scar or a tattoo that you get. You don't think: Oh, I have a tattoo all the time. At some point it's just […] yours’ (CM, f, 22). Furthermore, with growing self‐acceptance, self‐confidence also increased. One participant expressed her self‐acceptance as follows: ‘[…] It doesn't look pretty, but I've gotten used to it’ (CM, f, 61).

## 5 DISCUSSION

The aim of this study was to gain deeper insights into the differential, perceived disease‐related stigmatization among individuals with chronic skin diseases. While many studies examining stigmatization of chronic skin diseases only focus on one disease like psoriasis,^31^ another purpose of this study was to simultaneously observe a total of three diseases, namely psoriasis, AD and mastocytosis. Results show that individuals for all three diseases experienced stigmatization in all studied areas of life: in everyday life, at work, in sports and in relationships. Furthermore, participants expressed self‐stigmatization as well as individual growth through stigmatization. Negative self‐perception and self‐exclusion were present in all areas of life except for in relationships. Overall, negative self‐perception and self‐exclusion were most frequently mentioned across all dimensions. Furthermore, internal stigmatization can occur without being triggered by other people. Participants seem to be inclined to withdraw themselves from certain situations and to hide their skin. This withdrawal allows them to avoid being potentially stigmatized by others.

Most of the external stigmatization was experienced with strangers rather than with friends or partners. In relationships, most of the participants reported positive and supportive behaviour. External stigmatization by strangers occurred mostly through staring and insensitive or disrespectful behaviour and was especially prominent in daily life and sports. Another study showed similar results: the most troublesome aspect of feeling stigmatized was staring at the skin from strangers followed by people considering the skin disease contagious. This study, however, only examined a population of psoriasis‐patients, which limits the conclusions that can be made regarding other chronic skin diseases.^32^ Concerning external stigmatization, a recent review investigated this topic in visible skin diseases and concluded that further research is needed to examine causes and impacts of external stigmatization.^16^

Concerning stigmatization in the workplace, only a few participants reported negative experiences in this study. In contrast, a study examining stigmatization of individuals with AD found that more than half of participants expressed feelings of social stigmatization,^33^ which can also occur in professional contexts.

There seems to be a lack of social support from medical professionals for individuals with chronic skin diseases. Instead of receiving help or being listened to, participants in this study mostly felt that they were being ignored and that they were even being labelled hypochondriacs. According to literature, stigmatization can further lead to several psychological disorders.^9^, ^10^, ^11^, ^12^, ^34^ In particular, a lack of knowledge from general practitioners can delay the diagnosis of psychiatric conditions and hinder treatment.^35^ Furthermore, psoriasis patients who received a higher level of social support from medical professionals showed lower levels of depression, higher QoL, and more acceptance of life with the disease.^36^

Despite the negative impact of stigmatization due to chronic skin diseases, participants of this study experienced individual growth over time through positive internal change, increased self‐acceptance, and acceptance of the skin condition. This shows that healthy handling of stigmatization is possible. Further investigations in this area could demonstrate how patients could profit from more insight into the psychological, behavioural and social mechanisms leading to this positive development.

While stigmatization is often quantified using validated questionnaires^18^ like the Feelings of Stigmatization Questionnaire^37^ or the Internalized Stigma Scale (ISS),^38^ these questionnaires can be applied to all types of diseases, neglecting the specific nuances of chronic skin diseases. Although Dimitrov and Szepietwoski mention in their review^39^ that there is one stigmatization‐questionnaire specific for psoriasis^32^ and one for AD,^9^ dermatology‐specific questionnaires appear to be under‐used. Additionally, no questionnaire for mastocytosis could be found,^39^ which demonstrates a lack of assessment tools for stigmatization in masocytosis.

To date, this is the first qualitative study that could be found that examines stigmatization of the chronic skin diseases psoriasis, AD, and mastocytosis. One strength of this study is the focus on different areas of life instead of only assessing stigmatization in general. Moreover, the desire for social acceptance may be minimized by the usage of online interviews instead of face‐to‐face interviews.^40^ Online interviews can, however, also be considered a limiting factor because of the separation of interviewer and participant; feelings of distance might encourage self‐presentation and decrease authenticity.^41^ Another possible limitation is the lack of male participants for mastocytosis. Furthermore, recall bias and social desirability bias could have influenced the results.

In conclusion, this study suggests that people with chronic skin diseases experience stigmatization in different areas of life, namely at work, in sports, in relationships, and in interactions with medical professionals. Receiving better social support, for example, from medical professionals, could lower the mental burden and raise acceptance of living with the disease. Professionals in health‐related fields should advocate for and support broader systemic and policy efforts that seek to reduce stigmatization towards people with visible chronic skin diseases. As part of strengthening patient‐centred care and improving long‐term well‐being, more qualitative studies are needed to better understand the impact of stigmatization as well as to develop suitable questionnaires and interventions in the field of mental health and dermatology.

## CONFLICT OF INTERESTS

S. Ziehfreund has received speaker honoraria and travel fees from Novartis. M. C. Schielein has been an advisor and/or received speaker's honoraria of the following companies: Beiersdorf, Janssen‐Cilag, Leo Pharma, Novartis. A. Zink has been an advisor and/or received speaker's honoraria and/or received grants and/or participated in clinical trials of the following companies: AbbVie, Almirall, Amgen, Beiersdorf Dermo Medical, Celgene, Eli Lilly, GSK, Janssen Cilag, Leo Pharma, Miltenyi Biotec, Novartis, Sanofi‐Aventis, Takeda Pharma, UCB Pharma.

## AUTHOR CONTRIBUTIONS

**P. Heim‐Ohmayer**: Conceptualization (Equal); Data Curation (Equal); Formal analysis (Equal); Investigation (Equal); Methodology (Equal); Project Administration (Equal); Resources (Equal); Validation (Equal); Visualization (Equal); Writing – Original Draft (Equal); Writing – Review and Editing (Equal). **A. Freiberger**: Conceptualization (Equal); Data Curation (Equal); Investigation (Equal); Methodology (Equal); Resources (Equal); Validation (Equal); Visualization (Equal); Writing – Review and Editing (Equal). **M. Gedik**: Conceptualization (Equal); Data Curation (Equal); Investigation (Equal); Methodology (Equal); Resources (Equal); Validation (Equal); Visualization (Equal); Writing – Review and Editing (Equal). **J. Beckmann**: Conceptualization (Equal); Methodology (Equal); Project Administration (Equal); Supervision (Equal); Writing – Review and Editing (Equal). **S. Ziehfreund**: Conceptualization (Equal); Funding Acquisition (Equal); Methodology (Equal); Project Administration (Equal); Supervision (Equal); Validation (Equal); Writing – Review and Editing (Equal). **A. Zink**: Conceptualization (Equal); Funding Acquisition (Equal); Investigation (Equal); Project Administration (Equal); Resources (Equal); Supervision (Equal); Writing – Review and Editing (Equal). **W. Hähl**: Conceptualization (Equal); Formal analysis (Equal); Methodology (Equal); Project Administration (Equal); Resources (Equal); Supervision (Equal); Validation (Equal); Writing – Review and Editing (Equal). **M. Schielein**: Conceptualization (Equal); Data Curation (Equal); Funding Acquisition (Equal); Investigation (Equal); Methodology (Equal); Project Administration (Equal); Resources (Equal); Supervision (Equal); Validation (Equal); Writing – Review and Editing (Equal).

[Correction added on 23‐November‐2022, after first online publication: Author Contribution was added.]

## ETHICS STATEMENT

The study was conducted in accordance with the declaration of Helsinki and was reviewed and approved by the Ethics Committee at the Technical University Munich (reference 534/20 S). Written informed consent was obtained from all participants prior to each interview.

## Data Availability

The data that support the findings of this study are available from the corresponding author upon reasonable request.

## References

[1] Svensson A , Ofenloch RF , Bruze M , Naldi L , Cazzaniga S , Elsner P , et al. Prevalence of skin disease in a population‐based sample of adults from five European countries. Br J Dermatol. 2018;178(5):1111–8. 29247509 10.1111/bjd.16248

[2] Tizek L , Schielein MC , Seifert F , Biedermann T , Böhner A , Zink A . Skin diseases are more common than we think: screening results of an unreferred population at the Munich Oktoberfest. J Eur Acad Dermatol Venereol. 2019;33(7):1421–8. 30891839 10.1111/jdv.15494

[3] Mrowietz U , Schmid‐Ott G . Schuppenflechte – was sie schon immer über Psoriasis wissen wollten. Freiburg: Karger; 2012.

[4] Sabin BR , Peters N , Peters AT . Chapter 20: atopic dermatitis. Allergy Asthma Proc. 2012;33(Suppl 1):67–9. 22794693 10.2500/aap.2012.33.3553

[5] Matito A , Azaña JM , Torrelo A , Alvarez‐Twose I . Cutaneous mastocytosis in adults and children: new classification and prognostic factors. Immunol Allergy Clin North Am. 2018;38(3):351–63. 30007456 10.1016/j.iac.2018.04.001

[6] Ring J , Zink A , Arents BWM , Seitz IA , Mensing U , Schielein MC , et al. Atopic eczema: burden of disease and individual suffering – results from a large EU study in adults. J Eur Acad Dermatol Venereol. 2019;33(7):1331–40. 31002197 10.1111/jdv.15634

[7] Arents BWM , Mensing U , Seitz IA , Wettemann N , Fink‐Wagner AH , de Carlo G , et al. Atopic eczema score of emotional consequences—a questionnaire to assess emotional consequences of atopic eczema. Allergo J Int. 2019;28(7):277–88.

[8] Dalgard FJ , Svensson Å , Gieler U , Tomas‐Aragones L , Lien L , Poot F , et al. Dermatologists across Europe underestimate depression and anxiety: results from 3635 dermatological consultations. Br J Dermatol. 2018;179(2):464–70. 29247454 10.1111/bjd.16250

[9] Wittkowski A , Richards HL , Griffiths CE , Main CJ . The impact of psychological and clinical factors on quality of life in individuals with atopic dermatitis. J Psychosom Res. 2004;57(2):195–200. 15465076 10.1016/S0022-3999(03)00572-5

[10] Schuster B , Ziehfreund S , Albrecht H , Spinner CD , Biedermann T , Peifer C , et al. Happiness in dermatology: a holistic evaluation of the mental burden of skin diseases. J Eur Acad Dermatol Venereol. 2020;34(6):1331–9. 31838769 10.1111/jdv.16146

[11] Kim S , Lee JY , Oh JY , Chekal L , Lee DC . The association between atopic dermatitis and depressive symptoms in Korean adults: The Fifth Korea National Health and Nutrition Examination Survey, 2007‐2012. Korean J Fam Med. 2015;36(6):261–5. 26634090 10.4082/kjfm.2015.36.6.261 PMC4666859

[12] Zill JM , Christalle E , Tillenburg N , Mrowietz U , Augustin M , Härter M , et al. Effects of psychosocial interventions on patient‐reported outcomes in patients with psoriasis: a systematic review and meta‐analysis. Br J Dermatol. 2019;181(5):939–45. 30291741 10.1111/bjd.17272

[13] Ferreira BR , Pio‐Abreu JL , Reis JP , Figueiredo A , Figueiredo A . Analysis of the prevalence of mental disorders in psoriasis: the relevance of psychiatric assessment in dermatology. Psychiatr Danub. 2017;29(4):401–6. 29197196 10.24869/psyd.2017.401

[14] Ferreira BIRC , Abreu JLPDC , Reis JPGD , Figueiredo AMDC . Psoriasis and associated psychiatric disorders: a systematic review on etiopathogenesis and clinical correlation. J Clin Aesthet Dermatol. 2016;9(6):36–43. PMC4928455 27386050

[15] Sandhu JK , Wu KK , Bui T‐L , Armstrong AW . Association between atopic dermatitis and suicidality: a systematic review and meta‐analysis. JAMA Dermatol. 2019;155(2):178–87. 30540348 10.1001/jamadermatol.2018.4566 PMC6439544

[16] Germain N , Augustin M , François C , Legau K , Bogoeva N , Desroches M , et al. Stigma in visible skin diseases – a literature review and development of a conceptual model. J Eur Acad Dermatol Venereol. 2021;35(7):1493–504. 33428316 10.1111/jdv.17110

[17] WHO . Global report on psoriasis. World Health Organization; 2016. https://apps.who.int/iris/handle/10665/204417

[18] Schielein MC , Tizek L , Ziehfreund S , Sommer R , Biedermann T , Zink A . Stigmatization caused by hair loss – a systematic literature review. J DTSCH Dermatol Ges. 2020;18(12):1357–68. 10.1111/ddg.14234 33015951

[19] World Health Organization . WHO global strategy on people-centred and integrated health services: interim report. World Health Organization; 2015. https://apps.who.int/iris/handle/10665/155002

[20] Barbarot S , Auziere S , Gadkari A , Girolomoni G , Puig L , Simpson EL , et al. Epidemiology of atopic dermatitis in adults: results from an international survey. Allergy. 2018;73(6):1284–93. 29319189 10.1111/all.13401

[21] Cohen SS , Skovbo S , Vestergaard H , Kristensen T , Møller M , Bindslev‐Jensen C , et al. Epidemiology of systemic mastocytosis in Denmark. Br J Haematol. 2014;166(4):521–8. 24761987 10.1111/bjh.12916

[22] Horny HP , Sotlar K , Valent P , Hartmann K . Mastocytosis: a disease of the hematopoietic stem cell. Dtsch Arztebl Int. 2008;105(40):686–92. 19623287 10.3238/arztebl.2008.0686 PMC2696962

[23] Avena‐Woods C . Overview of atopic dermatitis. Am J Manag Care. 2017;23(8 Suppl):S115–23. 28978208

[24] Carter MC , Metcalfe DD , Komarow HD . Mastocytosis. Immunol Allergy Clin North Am. 2014;34(1):181–96. 24262698 10.1016/j.iac.2013.09.001 PMC3863935

[25] Valent P , Horny HP , Escribano L , Longley BJ , Li CY , Schwartz LB , et al. Diagnostic criteria and classification of mastocytosis: a consensus proposal. Leuk Res. 2001;25(7):603–25. 11377686 10.1016/s0145-2126(01)00038-8

[26] Tong A , Sainsbury P , Craig J . Consolidated criteria for reporting qualitative research (COREQ): a 32‐item checklist for interviews and focus groups. Int J Qual Health Care. 2007;19(6):349–57. 17872937 10.1093/intqhc/mzm042

[27] Sommer R , Augustin M , Mrowietz U , Topp J , Schäfer I , von Spreckelsen R . Stigmatisierungserleben bei Psoriasis – qualitative Analyse aus Sicht von Betroffenen, Angehörigen und Versorgern. Der Hautarzt. 2019;70(7):520–6. 31134287 10.1007/s00105-019-4411-y

[28] Helfferich C . Interviewplanung und Intervieworganisation. Die Qualität qualitativer Daten Manuel für die Durchführung qualitativer Interviews. 2. Wiesbaden: VS Verlag für Sozialwissenschaften; 2005. p. 147–73.

[29] Mayring P . Qualitative Inhaltsanalyse: Grundlagen und Techniken. Weinheim: Beltz; 2010.

[30] Brooks J , Wearden A . A critical evaluation of the use of Interpretative Phenomenological Analysis (IPA) in health psychology. Psychol Health. 2006;21:87–108.

[31] Dalgard FJ , Bewley A , Evers AW , Gieler U , Lien L , Sampogna F , et al. Stigmatisation and body image impairment in dermatological patients: protocol for an observational multicentre study in 16 European countries. BMJ Open. 2018;8(12): e024877. 10.1136/bmjopen-2018-024877 PMC6307615 30580274

[32] Hrehorów E , Salomon J , Matusiak L , Reich A , Szepietowski JC . Patients with psoriasis feel stigmatized. Acta Derm Venereol. 2012;92(1):67–72. 21879243 10.2340/00015555-1193

[33] Carvalho D , Aguiar P , Mendes‐Bastos P , Palma‐Carlos A , Freitas J , Ferrinho P . Quality of life and characterization of patients with atopic dermatitis in Portugal: The QUADEP Study. J Investig Allergol Clin Immunol. 2020;30(6):430–8. 10.18176/jiaci.0443 31530518

[34] Vermeiren MR , Kranenburg LW , van Daele PLA , Gerth van Wijk R , Hermans MAW . Psychological functioning and quality of life in patients with mastocytosis: a cross‐sectional study. Ann Allergy Asthma Immunol. 2020;124(4):373–8.e2. 31923542 10.1016/j.anai.2019.12.020

[35] Basavaraj KH , Navya MA , Rashmi R . Relevance of psychiatry in dermatology: present concepts. Indian J Psychiatry. 2010;52(3):270–5. 21180416 10.4103/0019-5545.70992 PMC2990831

[36] Janowski K , Steuden S , Pietrzak A , Krasowska D , Kaczmarek L , Gradus I , et al. Social support and adaptation to the disease in men and women with psoriasis. Arch Dermatol Res. 2012;304(6):421–32. 22456752 10.1007/s00403-012-1235-3 PMC3401292

[37] Kacar SD , Soyucok E , Bagcioglu E , Ozuguz P , Coskun KS , Asık AH , et al. The perceived stigma in patients with alopecia and mental disorder: a comparative study. Int J Trichology. 2016;8(3):135–40. 27625566 10.4103/0974-7753.189005 PMC5007920

[38] Temel A , Bozkurt S , Senol Y , Alpsoy E . Internalized stigma in patients with acne vulgaris, vitiligo, and alopecia areata. Turkish J Dermatol. 2019;13(3):109–16.

[39] Dimitrov D , Szepietowski JC . Instruments to assess stigmatization in dermatology. Postepy Hig Med Dosw. 2017;71(0):901–5. 10.5604/01.3001.0010.5607 29151062

[40] Oltmann S . Qualitative interviews: a methodological discussion of the interviewer and respondent contexts. Forum: Qualitative Social Research. 2016;17(2):1–16.

[41] Janghorban R , Roudsari RL , Taghipour A . Skype interviewing: the new generation of online synchronous interview in qualitative research. Int J Qual Stud Health Well‐Being. 2014;9(1):24152. 24746247 10.3402/qhw.v9.24152 PMC3991833

